# Acquisition of Cholera Within the United States

**DOI:** 10.1177/2324709620904204

**Published:** 2020-02-01

**Authors:** Ganesh Maniam, Emily N. Nguyen, John Scott Milton

**Affiliations:** 1 Texas Tech University Health Sciences Center, Amarillo, TX, USA

**Keywords:** vibriosis, *Vibrio cholerae*, cholera, toxigenic, toxins, internal medicine, infectious disease, recognition, treatment

## Abstract

Cholera has been woven into human history through numerous pandemics, with the most recent ongoing since 1961. Global rates of cholera continue to decline, but outbreaks continue to pose diagnostic challenges for clinicians, which delays initiation of treatment and prolongs the disease course. Despite millions of infections and thousands of deaths worldwide each year, cholera remains rare in the United States, with the few cases each year usually being the result of pathogen acquisition while the patient traveled abroad. This article presents a unique case of cholera acquired in the United States, which emphasizes the necessary vigilance of symptom recognition, in the context of appropriate clinical investigation, in ensuring that the patient had a full recovery. Cholera in the United States is exceedingly rare, yet effective diagnosis with early initiation of treatment is known to reduce mortality and shorten disease course. While other more common diagnoses must definitely be excluded first, it is important for cholera to be kept on the differential for patients presenting with treatment refractory, watery diarrhea causing hypotension. This case of a patient with a recent travel history to Hawaii and infection with cholera underscores the importance of investigative medicine and clinical expertise in optimizing patient care, even when presented with rare illnesses.

## Background

The etiologic agents of cholera is *Vibrio cholerae*, either serotype O1 or serotype O139.^1^ Other serotypes are known as *V cholerae* non-O1 non-O139 because they do not produce cholera toxin and therefore do not cause true cholera; instead, these nontoxigenic serotypes are more similar to other *Vibrio* species such as *Vibrio parahaemolyticus* or *Vibrio vulnificus*, in that they cause a similar diarrheal type of illness known as vibriosis, which is more similar to a gastroenteritis.^1^ Annually, there are millions of reported cholera cases, as well as thousands of reported fatalities.^2^ Despite the aims of the World Health Organization (WHO) to reduce cholera deaths by at least 90% before 2030, it is likely that this pathogen will continue to be a public health concern for the foreseeable future.^3^ While it is certainly true that endemic and resource-poor countries account for the majority of the global disease burden, this should not completely exclude cholera from the differential diagnosis when there is recent-onset, treatment-refractory, profuse, watery diarrhea in the United States.

## Case Presentation

A 58-year-old female presented with gradual-onset right lower quadrant abdominal pain with associated diarrhea, fever, nausea, and dysuria; travel history was notable for a recent trip to Hawaii, while past medical history was notable for Addison’s disease, Sjogren’s syndrome, and rheumatoid arthritis treated with corticosteroid immunosuppression. Patient was admitted to the hospital due to concern of pyelonephritis, appendicitis, and adrenal crisis—but workup for these etiologies was unremarkable after a few days of inpatient hospitalization and treatment with metronidazole, piperacillin-tazobactam, and ciprofloxacin. Laboratory values at this time indicated metabolic alkalosis with hypokalemia and hypocalcemia. A multiplex polymerase chain reaction (PCR) test of the patient’s stool returned positive for *V cholerae* and negative for every other tested stool pathogen; the stool panel was repeated due to the rarity of cholera acquired in the United States but confirmed the results. Stool culture was not done at the time due to lack of the media of choice; however, a stool specimen was collected and sent to the state health department for culture. Treatment was initiated with doxycycline and ciprofloxacin, after all other antibiotics were discontinued; supportive care was initiated at this time via aggressive intravenous fluid hydration. After a week of inpatient hospitalization, the laboratory values indicated normalization of serum electrolytes while the patient endorsed a complete resolution of her pain and diarrhea. Antibiotics were discontinued after a 7-day course of doxycycline and ciprofloxacin, and the patient was recommended for discharge.

## Discussion

The characteristics of *V cholerae* have been well studied in the literature, classically as a “comma-shaped” gram-negative and acid-labile rod, with flagellar motility that is crucial to intestinal infection and colonization.^2^ The cholera toxin is of particular interest, since its production via the *toxR* gene is what differentiates cholera from vibriosis; the cholera toxin is responsible for the dehydration and hypotension; and eventual hypoperfusion is responsible for the deadly mortality rate associated with this devastating infection.^4^ The other serotypes of cholera, most commonly serotypes O75 or O141, are referred to as *V cholerae* non-O1 non-O139, and are more common in the United States but are similar to their deadlier relatives in many ways.^1^ However, because these serotypes are less infectious and do not cause epidemics, epidemiologists use the term “vibriosis” to define these illnesses rather than “cholera.”^1^

The current global pandemic of cholera, which started in 1961, certainly affects some regions more than others. Asia and Africa have many endemic countries with seasonal recurrence or episodic outbreaks of cholera.^2^ Meanwhile, Haiti and Mexico are the only countries in the Americas to report endemic cholera, though the rest of North and South America has local serotypes of *V cholera* that produce vibriosis rather than cholera.^2^ In 2014, the Centers for Disease Control and Prevention (CDC) reported 4 total cholera infections in the United States, which were all acquired through travel to endemic countries.^5^ In 2015, the CDC reported 5 total cholera infections in the United States, of which 4 were travel-associated while one was due to consumption of imported raw shrimp from the Philippines.^5^ This trend continues into 2016 with 15 reported cases, then 2017 with 10 reported cases, and 2018 with 8 reported cases^5^; the vast majority of these cases were likewise travel-associated acquisitions. As of October 2019, there has only been 1 reported case of toxigenic *V cholera* serotypes O1 and O139 in the United States.^5^ Acquiring this disease in the United States is, therefore, exceedingly rare.

The primary mechanism of cholera transmission is the fecal-oral route via consumption of contaminated seafood or water.^1^ This fecal-oral route is especially effective given that profuse watery diarrhea allows for bacterial shedding to easily contaminate water sources in poorly sanitized conditions or areas with already limited water supplies.^2^ In the case discussed here, in which cholera was likely acquired within the United States, the source of infection is unknown. Possible sources of infection include the shellfish or raw tuna that the patient consumed during a trip to Hawaii 2 weeks prior to presentation; the exact origin of the seafood, whether it was imported or not, is unknown. The incubation period of cholera is known to be 1 to 5 days,^2^ and the delayed onset of presentation in this patient is unknown given that there are no case reports in the literature of an incubation period approximating 2 weeks. It is also possible that this rare case of cholera acquired in the United States may have been more likely due to the immunocompromised state of the patient.

Infection with cholera presents as a profuse “rice-water” diarrhea with associated vomiting that can lead to death secondary to hypoperfusion within 12 hours of the initial onset of symptomatology,^5,6^ and this severe dehydrating illness is known as “cholera gravis.”^7^ However, the time from infection with *V cholera* to initial symptoms typically ranges from a few hours to 5 days, with reported median incubation period of toxigenic cholera to be 1.4 days.^8^ Therefore, the presentation of cholera symptoms 2 weeks later, as in this case, is atypical. Cholera must be contrasted against vibriosis, which is an uncommon infection that is increasing in the United States.^9^ Vibriosis is much more likely to present with gastroenteritis with an associated watery diarrhea, and these illnesses are mild to moderate in severity—such that these self-limiting illness often do not require medical attention; however, microbiology laboratory assistance and testing is often needed to differentiate these illnesses.^9^ In regions where cholera is not endemic, the diagnosis of cholera is often delayed due to clinician lack of familiarity with symptoms, which notoriously occurred during the 2010 Haiti cholera epidemic.^9^

Diagnosis of cholera is via recognition of clinical symptoms with a compatible patient history, and then confirmatory laboratory testing for *V cholerae*.^9^ In regard to this case, the rarity of cholera acquired in the United States certainly raises appropriate questions regarding the validity of serological testing. CDC guidelines stipulate that culture of a stool specimen remains the gold standard for laboratory diagnosis of cholera; however, it also states that suspected cases can be confirmed via either culture or PCR. In the case of this patient, the state health department laboratory was unable to isolate *V cholerae* from the stool specimen. The quality of the specimen may have been affected by several factors; the stool specimen was collected several days after initiation of antibiotics and was transported several hundreds of miles to the state health department on Cary Blair transport media.

While the culture result returned negative several weeks after the fact, the diagnosis was made using PCR testing. The most common diagnostic test in the United States is some form of a PCR assay, which is advantageous for its accuracy, and ability to detect even the smallest amounts of pathogen in stool samples. This patient was diagnosed using a FilmArray gastrointestinal pathogen panel, which consists of a multiplex PCR test of a stool sample for 22 common gastroenteritis-causing pathogens, including *V cholerae*. Multiplex PCR tests have been shown to have a 100% sensitivity and 95% specificity for detecting *V cholerae* O1 and O139 serogroups compared with routine stool culture, with an accuracy of 96%, positive predictive value of 90%, and negative predictive value of 100%.^6^ The FilmArray panel itself has been shown to have 100% specificity (95% confidence interval [CI] = 98-100) for *V cholerae*, and 100% specificity (95% CI = 51.1-100) for *Vibrio* species, compared with routine bacterial culture.^10^ Multiplex PCR tests like FilmArray target the specific gene sequences that are involved in producing cholera toxin, such as the ctxA amplicon; this affords considerable accuracy and specificity.^6^ Despite the rarity of cholera in the United States, 2 positive test results via the FilmArray gastrointestinal pathogen panel, especially in the context of patient history and presentation, are unlikely to be false positives.

The most important intervention in the treatment of cholera remains aggressive fluid rehydration, which has been demonstrated to reduce mortality to less than 0.5%.^11^ Concurrent antibiotic therapy is known to reduce the duration of diarrhea, and current WHO guidelines suggest antibiotic therapy for dehydrated patients, though there are other international guidelines that suggest antibiotic therapy for diagnosed cholera.^11^ With regard to treatment regimens, doxycycline is considered to be first-line therapy and ciprofloxacin is second-line therapy.^11^ Other treatment options include macrolides and cotrimoxazole.^11^

Access to clean water and increased sanitation are paramount to the prevention of cholera.^2,4,5^ The WHO aims to reduce cholera deaths by at least 90% before 2030, and their eradication efforts have been assisted by an oral cholera vaccination.^3^ The vaccine is approved by the WHO for endemic countries, but an isolated case of cholera acquisition in an immunocompromised patient does not warrant widespread vaccination of Hawaiian tourists or increased water safety regulations for the state.

In the United States, vibriosis is uncommon and cholera is rare, thus prevention and treatment falls on clinicians. Physicians should educate their patients traveling abroad to ensure that they have access to clean water and sanitized conditions. The tropical nature of Hawaii perhaps increases the risk of cholera acquisition, but there do not appear to be any studies in the literature regarding this topic. Additionally, individuals who are immunocompromised should be especially careful in water and food intake during any travel, taking care to avoid raw or undercooked meats—as this may put them at increased risk for infections such as cholera. Physicians or travel medicine specialists should counsel these individuals prior to their travel. When presented with a severe diarrheal illness of unknown etiology that is seemingly treatment refractory, it is necessary to keep cholera on the differential, though unlikely, in order to optimize patient care in the face of this historically deadly disease.

## Conclusion

Recognition and treatment of cholera in the United States is complicated due to its rarity, yet effective diagnosis with early initiation of treatment is known to reduce mortality and shorten disease course. While other more common diagnoses must definitely be excluded first, it is important for cholera to be kept on the differential for patients presenting with treatment refractory, watery diarrhea causing hypotension. This case of a patient with a recent travel history to Hawaii and developing cholera emphasizes the necessary vigilance of symptom recognition, in the context of appropriate clinical investigation, in ensuring the patient had a fully recovery.
